# Oral myiasis caused by *Chrysomya bezziana*

**DOI:** 10.4103/0973-029X.64304

**Published:** 2010

**Authors:** Leena S Sankari, K Ramakrishnan

**Affiliations:** *Department of Oral Pathology, Adhiparasakthi Dental College and Hospital, Melmaruvathur, Kancheepuram - 603 319, Tamil Nadu, India*

**Keywords:** *Chrysomya bezziana*, fly larvae, myiasis

## Abstract

Oral myiasis is a rare disease caused by larvae of certain dipteran flies. It is mostly reported in developing countries and in the tropics. Herein, a case of oral myiasis in the maxillary anterior region of a 14-year-old mentally challenged boy is being reported. The myiasis was caused by the larvae of *Chrysomya bezziana* species. The clinical findings are presented. Etiology and the importance of oral health in special people are also discussed.

## INTRODUCTION

Myiasis was first described by F. W. Hope in 1840. Myiasis is derived from the Greek word “myia,” meaning fly and “asis,” meaning disease.[1–3] Myiasis is caused by dipterous larvae that feed on the host dead or living tissues, liquid body substances or ingested food.[4,5] Myiasis frequently occurs in rural areas, infecting livestock, and in humans prevails in unhealthy individuals in third world countries.[2] Incidence of oral myiasis is comparatively lesser than that of cutaneous myiasis as oral tissues are not permanently exposed to the external environment.[3] Cases of oral myiasis have been reported to occur following dental extraction, nosocomial infection, in drug addicts, visits to tropical countries, in psychiatric patients[4,5] and conditions that are likely to cause prolonged mouth opening, like mouth breathing during sleep, senility, alcoholism[6] and mental retardation. The flies are attracted to the bad mouth odor due to neglected oral hygiene or fermenting food debris. Persistent mouth opening facilitates the deposition of the eggs by the adult fly,[4] with India’s subtropical climate conducive to their breeding.[7]

## CASE REPORT

**Figure 1:**
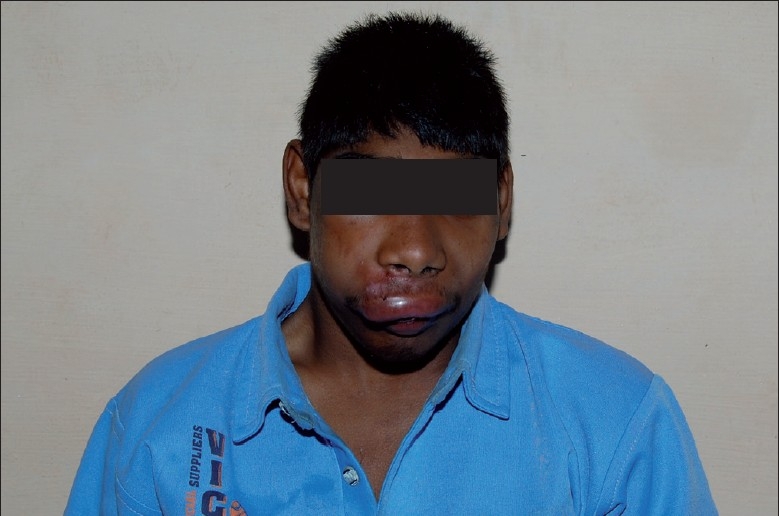
Appearance of the patient at the time of presentation

A 14-year-old mentally challenged boy presented to our hospital with a swelling of the upper lip and the right side of his face for the past 3 days [Figure 1]. His parents accompanied him. The patient had a history of fits 10 days back following which he sustained fracture of the upper front teeth. Following extraction of the fractured tooth, he developed a wound in the same location that started to increase in size. He had severe and continuous pain over the wound. The parents also noticed worms in the mouth. The patient also has a history of seizures for which he is under medication. The patient was of a low socioeconomic status, poorly built, apprehensive and febrile. Diffuse swelling was seen over the right side of the face. Periorbital edema was present and the upper lip was swollen.

**Figure 2:**
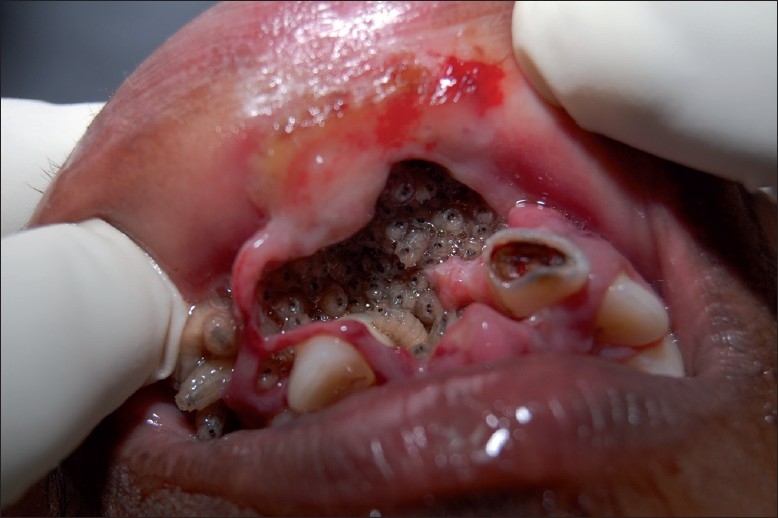
Maggots in the maxillary anterior region

Intraoral examination revealed a necrotic area in the maxillary anterior region involving the vestibular sulcus and labial mucosa extending from the right canine to the left canine, measuring 6 cm × 4 cm. A number of maggots were seen in the necrotic area [Figure 2]. The surrounding area was erythematous and swollen. The patient had poor oral hygiene. The teeth in the necrotic area 12 and 21 exhibited significant mobility, and 21 was decayed. Based on the clinical findings, the case was diagnosed as oral myiasis. Hematological analysis was within normal limits.

**Figure 3:**
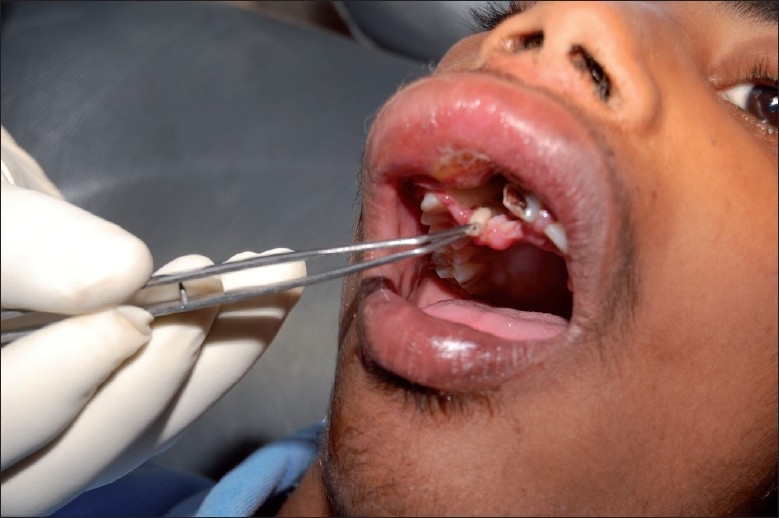
Removal of maggots with a tweezer

**Figure 4:**
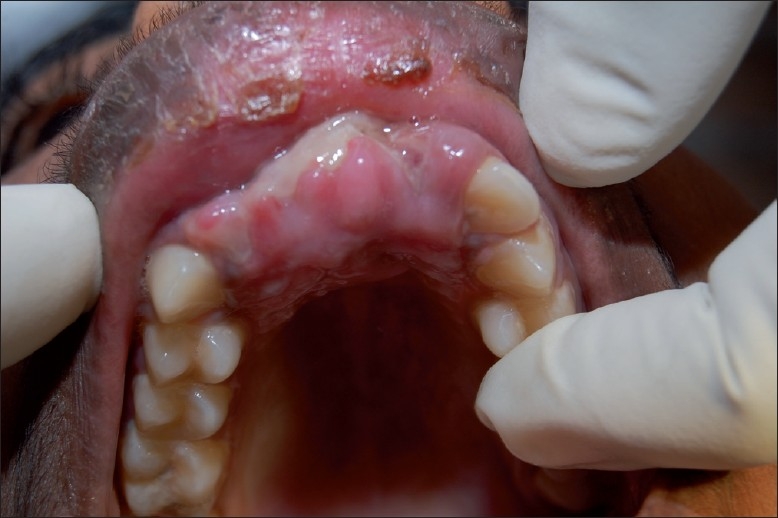
Healing of the tissues after removal of larvae

The treatment was performed by oral and maxillofacial surgeons of our hospital. The patient was hospitalized for 3 days. The treatment included flushing the affected area with turpentine oil followed by the administration of local anesthesia and manual removal of maggots with tweezers [Figure 3]. Around 50–60 maggots were harvested. Teeth 12 and 21 were extracted. The area was then washed with saline, followed by irrigation with betadine. Broad spectrum antibiotics amoxycillin with clavaualnic acid and ibuprofen with paracetamol were prescribed. This procedure was repeated again until the maggots were completely removed. The next day the edema had subsided considerably. The parents were given extensive environmental and personal hygiene instructions. The maggots were sent to a Veterinary Parasitology Department in Chennai Veterinary College for identification. The maggots were 12–15-mm long, whitish and without obvious body processes. The peritreme of the posterior spiracle was open and the anterior spiracle had four to six lobes. These features were compatible with *Chrysomya bezziana* larvae. The larvae were thus identified as *Chrysomya bezziana* species. The wound was left open to heal by secondary intention [Figure 4]. The patient was reviewed 1 week later and the results were satisfactory.

## DISCUSSION

Myiasis is defined as infestation of live human and vertebrate animals with dipterous larvae that feed on the host’s dead or living tissue, liquid body substances or ingested food.[2,4,8,9] They infest several parts of the body as in cutaneous, ophthalmic, oral, urogenital, nasopharyngeal and intestinal myiasis.[6]

Low socioeconomic status, immunocompromised state, debilitated and unhygienic living conditions are the main contributing factors responsible for myiasis. The risk factors for oral myiasis include suppurative lesions, facial trauma, mouth-breathers, extraction wounds, fungating carcinomas and others conditions.[2,3,6]

In our case, the extraction wound was the most probable cause in the development of myiasis. Lack of personal hygiene, communicating ability and negligence of the caretaker might have led to delayed presentation.

Flies causing myiasis belong to the order Diptera.[10,11] The genera commonly reported are Sarcophagidae, Calliphoridae, Oestridae and Muscidae from the Diptera order.[8] *Chrysomya bezziana*, the Old World screw-worm fly, belongs to the genera Calliphoridae. It is an obligatory myiasis producer whose larvae develop only in living tissue,[9,12] and human cases of *Chrysoma bezziana* infestations are uncommon.[13] The species was first found in animal wounds in Hong Kong in the year 2000.[9,13] The first case in humans was documented in 2003 in Hong Kong.[13] *Chrysomya bezziana* is widely distributed throughout Asia, including China and neighboring regions of Hong Kong, such as Guangdong, Guangxi, Yunnan and Taiwan. It is also found in tropical Africa, the Indian subcontinent and Papua New Guinea.[9,12,13]

The adult female lays eggs on live mammals. The sites of infestation are usually superficial wounds, open sores and mucous membranes in body orifices such as the mouth, ear and nose.[14] The eggs hatch within 24 h and the resulting larvae burrow into the host’s tissues head-downwards into the wound in a characteristic screw-like fashion, feeding on living tissue. The larvae release toxins to destroy the host tissue. Proteolytic enzymes released by the surrounding bacteria decompose the tissue on which the larvae feed.[2,4,11] The larvae complete their development in 5–7 days. They then wriggle out of the wound and fall to the ground to pupate.[5,9]

The treatment consists of topical application of turpentine oil, mineral oil, chloroform, ethyl chloride or mercuric chloride followed by manual removal of the larvae and surgical debridement.[4] Recently, a systemic treatment with Ivermectin, a semisynthetic macrolide antibiotic isolated from *Streptomyces avermitilis*[1] has been used for the treatment of oral myiasis.[2,8] The cases of oral myiasis with no medical systemic complications recover completely on removal of larvae.[15]

Infestations with *Chrysomya bezziana* differ from other maggot infestations because there is tissue invasion even in the absence of any pre-existing necrotic tissue. The *Chrysomya bezziana* maggots may cause serious and permanent tissue damage and extremely infested wounds can even lead to death in the absence of proper treatment. It is usually the special people with mental or physical disability who are affected because of the difficulties in maintaining good oral hygiene due to poor manual dexterity and negligence of parents/guardians.[4] Therefore, it becomes necessary for these patients to be exposed to dental examination at regular intervals to prevent such diseases. The personnel taking care of special people are advised to ensure personal hygiene and adopt suitable practices for a good environmental hygiene to prevent the occurrence of infestations.
